# No gene by stressful life events interaction on individual differences in adults’ self-control

**DOI:** 10.3389/fpsyt.2024.1388264

**Published:** 2024-04-17

**Authors:** Yayouk Eva Willems, Laurel Raffington, Lannie Ligthart, Rene Pool, Jouke Jan Hottenga, Catrin Finkenauer, Meike Bartels

**Affiliations:** ^1^ Max Planck Institute for Human Development, Max Planck Research Group Biosocial – Biology, Social Disparities, and Development, Berlin, Germany; ^2^ Department of Biological Psychology, Vrije Universiteit Amsterdam, Amsterdam, Netherlands; ^3^ Department of Interdisciplinary Social Science, Universiteit Utrecht, Utrecht, Netherlands; ^4^ Amsterdam Public Health Research Institute, Amsterdam University Medical Centres, Amsterdam, Netherlands

**Keywords:** self-control, genetics, stress, gene-environment interaction, polygenic scores

## Abstract

**Background:**

Difficulty with self-control, or the ability to alter impulses and behavior in a goal-directed way, predicts interpersonal conflict, lower socioeconomic attainments, and more adverse health outcomes. Etiological understanding, and intervention for low self-control is, therefore, a public health goal. A prominent developmental theory proposes that individuals with high genetic propensity for low self-control that are also exposed to stressful environments may be most at-risk of low levels of self-control. Here we examine if polygenic measures associated with behaviors marked by low self-control interact with stressful life events in predicting self-control.

**Methods:**

Leveraging molecular data from a large population-based Dutch sample (N = 7,090, Mage = 41.2) to test for effects of genetics (i.e., polygenic scores for ADHD and aggression), stressful life events (e.g., traffic accident, violent assault, financial problems), and a gene-by-stress interaction on self-control (measured with the ASEBA Self-Control Scale).

**Results:**

Both genetics (β =.03 -.04, p <.001) and stressful life events (β = .11 -.14, p <.001) were associated with individual differences in self-control. We find no evidence of a gene-by-stressful life events interaction on individual differences in adults’ self-control.

**Conclusion:**

Our findings are consistent with the notion that genetic influences and stressful life events exert largely independent effects on adult self-control. However, the small effect sizes of polygenic scores increases the likelihood of null results. Genetically-informed longitudinal research in large samples can further inform the etiology of individual differences in self-control from early childhood into later adulthood and its downstream implications for public health.

## Introduction

Difficulties with self-control, including the ability to delay gratification, control impulses, and regulate emotions, predicts interpersonal conflict, criminal involvement and more adverse health outcomes. For example, adults with lower self-control are more likely to experience workplace and interpersonal conflicts, display unhealthier lifestyles, experience mental health problems, show faster biological aging, and a shorter health span than individuals with higher self-control (1–4). Understanding the etiology, early detection, and intervention of low self-control in adulthood is, therefore, a public health goal.

Family studies suggest that individual differences in self-control are influenced by both genetic and environmental effects (5, 6). Stressful life events, such as exposure to violence, interpersonal conflict, and economic hardships, are commonly associated with reduced self-control both in the short-term and the long-term by draining psychological reserves needed for self-control and developmentally by chronic physiological insults or impacted learning (7–10).

**Figure 1:**
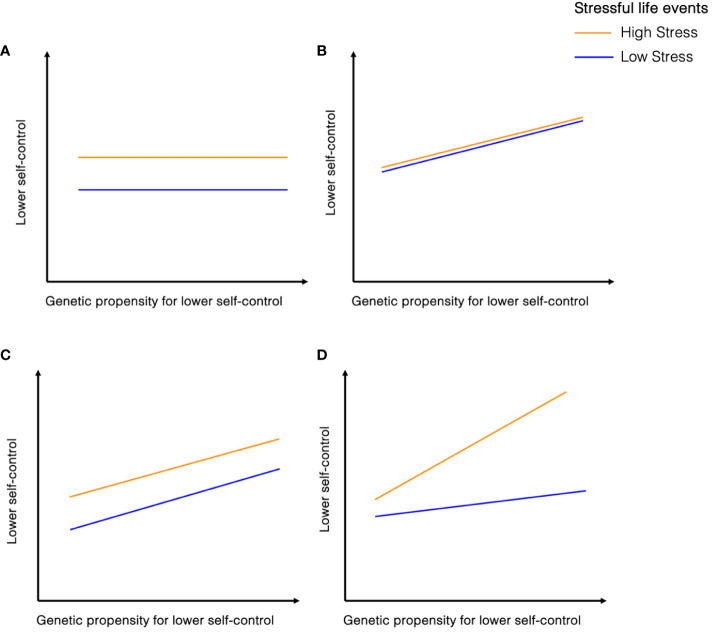
Hypothesized relationships between genetic propensity for lower self-control and stressful life events on individual differences in self-control. **(A)** reflects a significant effect of stressful life events on self-control, with individuals having experienced more life stressors showing lower self-control. there are no effects of genetic propensity on self-control. **(B)** indicates a significant effect of genetic propensity on self-control, with those with a higher polygenic score for aggression or ADHD showing lower self-control. there are no effects of stressful life events on self-control. **(C)** reflects a significant effect both of stressful life events and genetic propensity on self-control. Those who experienced more life stressors show lower self-control, and those who have higher polygenic scores for ADHD or aggression show lower self-control. This does not indicate an interaction effect, as the joint effects of genetic and environmental risk factors are not significantly greater than the sum of the separate effects. **(D)** displays one potential type of gene-environment interaction as posited by diathesis stress model, where those who have both experienced more stressful life events *and* have a higher polygenic score for ADHD or aggression show elevated self-control problems. Other gene-environment interactions are possible, *e.g.* buffering effect which are not illustrated here.

A prominent developmental theory proposes that individuals with high genetic propensity for low self-control that are also exposed to stressful environments may be most at-risk of low levels of self-control. The diathesis-stress model, proposes that stress may activate or increase a vulnerability (a diathesis) that transforms the potential of vulnerability into the actuality of psychopathology (11, 12). This can also be operationalized as gene-by-environment interaction (G x E), where different genotypes induce different sensitivity to the environment (13, 14). Extending this theoretical framework to self-control, it is hypothesized that the genetic vulnerability for low self-control interacts with the experience of life stressors to elevate the risk of developing self-control problems (15, 16, see **Figure 1**).

Thus far, attempts to test this hypothesis mostly pertained to candidate gene studies and yield inconclusive results (17, 18). The latest advances in genomics have enabled a more comprehensive understanding of complex behaviors going beyond candidate genes and acknowledging their polygenic nature (19, 20). This is, amongst others, achieved through the use of polygenic scores, which aggregate the combined effects of various genetic variants linked to a specific trait (21). However, few studies so far have used this polygenic approach when investigating G x E effects on self-control. Leveraging data from a large population-based adult sample (N=7,090), we test for effects of genetics (i.e., polygenic scores of attention-deficit hyperactivity disorder and aggression), stressful life events (e.g., traffic accident, violent assault, financial problems), and a gene-by-stress interaction on adult self-control (see **Figure 1**).

## Methods

### Participants

This study is based on data of participants of the Netherlands Twin Register (NTR). The NTR was initiated in 1987, and collects data on the health and wellbeing of twins and their family members across the lifespan (22). We used an adult subsample of the NTR, with data available on self-control, experienced stressful life events, and genotype (N=7,090, M_age_ = 41.2, SD_age_ = 15.4, 18-90 years old, 66% female, all European Ancestry). All participants provided written informed consent, and the data collection was approved by the Central Ethics Committee on Research Involving Human Subjects of the VU University Medical Centre, Amsterdam.

## Measures

### Self-control

We used the ASEBA Self-Control Scale (ASCS) to assess self-control (23). This self-reported questionnaire consists of 8 items assessing varying dimensions of self-control based on selected items of the ADHD and Aggression subscale of the Child Behavior Checklist (24). Items include: “I fail to finish things that I should do”, “I can’t concentrate, can’t pay attention for long”, I break rules at work or elsewhere”, “I am impulsive or act without thinking”, “I am easily distracted”, “I am sullen, or irritable”, “I have sudden changes in moods or feelings”, “I can have a hot temper” (see 23 for a thorough discussion of item selection). Items are measured on a 3-point scale (1= Not true, 2= Somewhat or Sometimes True, 3= Very True or Often True). We created a sum score across these 8 items, so that higher scores reflect lower self-control. The scale shows good inter-rater and test-retest reliability (23), and has been applied across several other cohorts (25, 26).

### Stressful life events

We used the Dutch Life Event Scale (“Schokverwerkings Inventarisatie Lijst”, 27) to assess the experience of stressful life events across the lifespan. This measure has been used earlier in the same data to assess the effect of life stressors on psychopathology within the Netherlands Twin Register (28, 29). It consisted of 11 items about stressful life events including having experienced traffic accident, violent assault, sexual assault, robbery, serious illness or injury of self or a significant other (e.g. partner, child, parent), death of a significant other, dismissal from work, financial problems, and relationship problems with a close partner. Response categories were 1= Not experienced, 2= Experienced Less than a year ago, 3= Experienced 1-5 years ago, 4= Experienced longer than 5 years ago. We considered both lifetime and past year stressful experiences respectively to understand the cumulative impact of stress and the acute influence of recent events on individual’s self-control problems. As such, we created two continuous scores; one sum score for stressful life events experienced in the previous year (SLE previous year) and one sum score for experience of stressful life events across the lifespan (SLE lifetime).

### Polygenic scores

Genome Wide Association Studies (GWAS) use genetic data of large samples to detect the cumulative effects of single nucleotide polymorphisms (SNPs) on an outcome of interest. Using such summary statistics of GWAS allows to create an individual polygenic score for all genotyped participants, weighing the predictive value of each individual SNPs and generating an overall predictive polygenic score (21).

While there have been efforts to identify SNPs associated with self-control related traits, the summary results from GWASs of impulsivity and delay of gratification are not publicly available (30, 31). We therefore created polygenic scores based on traits that reflect low self-control behaviors, namely the summary results from GWASs of attention-deficit hyperactivity disorder (ADHD, 32) and aggression (33). ADHD taps into struggles with impulsivity and difficulty regulating one’s emotions and behavior (34, 35). Similarly, aggression taps into issues regulating impulses and emotions that ultimately lead to aggressive behaviors (35, 36). Both the PGI for attention problems and aggression problems and our measure of self-control indexes impulsive and emotional aspects of self-control reflected in questions on aggression and attention problems subscale (23, 32, 33).

Preprocessing of the genetic data is described in **Supplementary Material 1**. As the genotyped participants of the NTR were part of the ADHD and aggression GWA studies, we used summary statistics where the NTR participants were left out to avoid an overestimation of the effects. LD-pred (v0.9) was used to compute the polygenic scores in the target sample, accounting for linkage disequilibrium (LD) among SNPs by using the LD structure of a set of well-imputed variants in a selection of unrelated individuals in the NTR sample (37). Both selections were performed on the genotype data described above. The fraction of causal SNPs was set at 0.50 as this was previously shown to perform optimally in the NTR population (33). Setting it to infinity showed similar results.

### Statistical analyses

Regression analyses were carried out with self-control as the dependent variable, using generalized estimation equations (GEE) in R clustering on family membership to adjust for dependency of the observations (version 4.4.2, 38, using package “geepack”, 39). We added age, age2, sex, 10 principal components, and array as covariates in the regression analyses. Scores were log-transformed to account for skew. We included 10 principal components of genetic similarity to ancestral reference groups to account for population stratification and/or cryptic relatedness. These 10 principle components refer to the linear combinations of genotypes of SNPs, which, when included, reduce the bias of confounding (40). In case of significant interaction effect, we would include interaction-terms of covariates x genes (e.g., age x polygenic score) and covariates x environment (e.g., age x stressful life event) to eliminate effects of interactions between covariates and the variables of interest (41). We conducted the analyses separately for having experienced stressful life events less than a year ago (SLE previous year) and having experiences stressful events across the life time (SLE lifetime). We also conducted the analyses separately for the polygenic score of ADHD and the polygenic score of aggression. We applied Bonferroni-type adjustment to correct for inflated Type 1 error due to multiple testing (taking alpha level of 0.05/8 tests = .006). The results section presents standardized beta estimates, their 95% confidence interval, and p-values of the main and interaction effects. Descriptives can be found in **Supplementary Material 2**.

## Results

We first examined whether genetic influences were associated with individual differences in self-control by regressing self-control on polygenic score and covariates. We found that both the polygenic score for ADHD and aggression were associated with lower self-control, but effect sizes were very small (PGS_ADHD_ *β* =.03, SD=.01, 95% CI.02 -.06, *p* <.001; PGS_aggression_ *β* = .04, SD=.01, 95% CI.02 -.07, *p* <.001). The PGI for ADHD and aggression were modestly correlated (*r*=.20, *p*<.001), which is in line with the notion that self-control is a broad construct influenced by a variety of underlying genetic factors (35).

Second, we examined whether stressful life events were associated with self-control by regressing self-control on life events and covariates. We found that having experienced more stressful life events (SLE) was associated with lower self-control (SLE last year: *β* = .11, SD=.01, 95% CI.09 -.15, *p* <.001; SLE lifetime: *β* = .14, SD=.01, 95% CI.11 -.16, *p* <.001).

**Figure 2:**
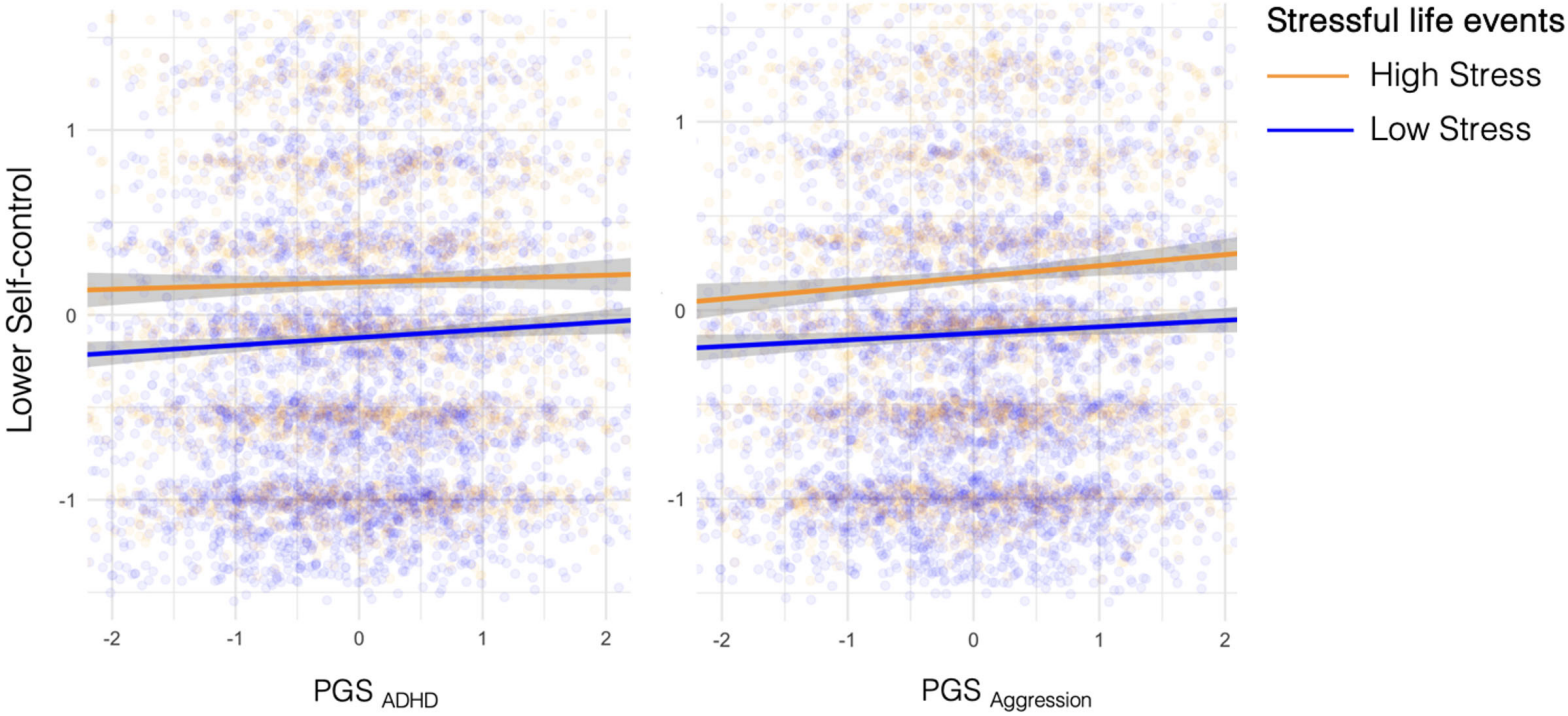
Association between PGS for self-control problems and self-control. Stressful life events represent stressful life events experienced *across the lifetime*. For illustration purposes, participants were stratified into a high stress and low stress group by mean split of stressful life events (M_stressfull life events_= 2.5). The lines for the two groups are approximately parallel and resemble those in Panel C in **Figure 1**, indicating there is a main effect of stressful life events and genetic propensity on self-control problems, but no significant interaction effect between the two.

Third, we examined whether there was a gene by stressful life events interaction on self-control by regressing self-control on polygenic score, stressful life events, polygenic score by stressful life events interaction, and covariates. We did not find evidence for a gene-environment interaction (see **Table 1**; **Figure 2**).

**Table 1:** Coefficients of the interaction effects of stressful life events (SLE) and polygenic scores (PGS) associated with self-control.

	*β*	SE	95% CI	*p*-value
*Model 1: PGS ADHD & SLE previous year*				
SLE Previous year	.11	.01	.09 -.15	<.001
PGS ADHD	.03	.01	.02 -.06	<.001
PGS ADHD * SLE previous year	.00	.01	-.02 -.03	.54
*Model 2: PGS Aggression & SLE previous year*				
SLE Previous year	.12	.01	.09 -.15	<.001
PGS Aggression	.04	.01	.02 -.07	<.001
PGS Aggression * SLE previous year	.01	.01	-.02 -.04	.48
*Model 3: PGS ADHD & SLE lifetime*				
SLE lifetime	.15	.01	.13 -.17	<.001
PGS ADHD	.03	.01	.02 -.06	<.001
PGS ADHD * SLE lifetime	-.02	.01	-.05 -.00	.07
*Model 4: PGS Aggression & SLE lifetime*				
SLE lifetime	.15	.01	.13 -.17	<.001
PGS Aggression	.04	.01	.02 -.07	<.001
PGS Aggression * SLE lifetime	.01	.02	-.02 -.04	.48

All analyses included age, age^2^, sex, 10 principal components, and array as covariates. Analyses were conducted separately for each polygenic score (PGS), respectively.

## Discussion

The diathesis-stress theory hypothesizes that peoples’ genetic propensity interacts with environmental stressors as a shaping factor for the development of behavioral problems (11, 12). Using data from a large adult population-based sample (N=7,090, Mage = 41.2, SD_age_ = 15.4, 66% female), we investigated this hypothesis for self-control. We found that participants who had experienced more life stressors showed lower levels of self-control. We also found that participants with higher genetic propensity for self-control problems (using the polygenic score for ADHD and aggression, respectively), showed lower levels of self-control. However, we did not find a significant gene-by-environment (G x E) interaction effect: The joint effects of genetic and environmental risk factors were not significantly greater than the sum of the separate effects.

We confirm previous findings that individual differences in self-control are related to both genetic variation and stressful life events (6). We extend these findings by showing that polygenic scores based on large GWAS for ADHD and aggression are associated with low self-control. This suggest that these scores are beneficial for future research intended to explore or adjust for genetic influences on individual variations in self-control. Additionally, in line with earlier research (8, 10, 42), we find that having experienced life stressors in the past year or across the lifetime are both associated with lower levels of self-control in adulthood.

Our findings are consistent with the notion that genetic influences and stressful life events exert largely independent effects on adult self-control. While some studies do find significant G x E effects (43, 44), other studies fail to detect G x E moderation effects on self-control related traits (e.g. 45–48). This and other research (e.g., 49) suggest that consistently identifying gene-environment (G x E) interactions for the prediction of complex traits, which are shaped by numerous genetic and environmental factors, is challenging. In the future, it is recommended to rethink how to select and measure relevant environmental measures (49) or to create more fine-tuned polygenic scores for example by identify genetic variants associated with variation in the outcome rather than the average level (49, 50) or to conduct genome-wide by environment interaction studies (GWEIS) on the genomic level to better capture gene-environment interactions (51).

The results of the current study should be interpreted with some limitations in mind. First, the small effect sizes of the polygenic scores increase the likelihood of the G x E null results. We utilized polygenic scores for ADHD and aggression, which do not completely represent the broader dimensions of self-controlling capacities (35, 52) and are likely to partly explain the low effect sizes as found in other studies (e.g., 4). One future recommendation would be to apply Genomic Structural Equation Modeling (53) to model multivariate genetic associations among self-control related phenotypes to generate a more encompassing polygenic score of self-control (e.g. also including GWAS results of impulsive personality, delay discounting, and executive functioning, 30, 31, 54).

Second, the timing of gene-environment interactions can be developmentally specific, and G x E interactions might have more noticeable or lasting impacts during early childhood compared to adulthood. Future research is recommended to replicate our findings in a pediatric sample.

Lastly, our life events measure only included stressful life events (e.g., theft, illness, financial strain etc.) and not positive life events (e.g., marriage, birth of a child) which can also impact people’s self-control levels (55, 56). We additionally weighted all stressful life events to be the same, although some stressful life events could be more strongly associated with low self-control than others. Future research taking more fine-grained approach to assessing life events could provide interesting avenues.

To conclude, this study sought to understand individual differences in self-control by examining the interplay between genetic factors and life stressors. The findings showed that while genetic scores for ADHD and aggression and life stress independently predicted self-control levels, their combined influence did not significantly exceed their individual effects, suggesting no gene-environment interaction. The results highlight the importance of both genetics and life stress in understanding self-control, and pointing to the need for further research to unravel their complex relationship.

## Data availability statement

The data analyzed in this study is subject to the following licenses/restrictions: genetic data. Requests to access these datasets should be directed to https://tweelingenregister.vu.nl/.

## Ethics statement

Data collection complied with current APA Ethical Principles of Psychologists and Code of Conduct and was approved by the medical ethical review committee of the VU Medical Center Amsterdam (NTR25052007). The studies were conducted in accordance with the local legislation and institutional requirements. Written informed consent for participation was not required from the participants or the participants’ legal guardians/next of kin in accordance with the national legislation and institutional requirements.

## Author contributions

YW: Conceptualization, Data curation, Formal analysis, Investigation, Methodology, Project administration, Visualization, Writing – original draft, Writing – review & editing. LR: Writing – review & editing. LL: Data curation, Project administration, Writing – review & editing. RP: Data curation, Formal analysis, Writing – review & editing. JH: Data curation, Formal analysis, Writing – review & editing. CF: Conceptualization, Supervision, Writing – review & editing. MB: Conceptualization, Supervision, Writing – review & editing.
