# Supplementary material for: No gene by stressful life events interaction on individual differences in adults’ self-control
Source: Front Psychiatry. 2024 Apr 17;15:1388264. doi: 10.3389/fpsyt.2024.1388264 (PMC11061522; doi:10.3389/fpsyt.2024.1388264)
Supplement: Supplementary file 1 [file DataSheet_1.docx]

**Supplemental Material**

Table of Contents

[Supplement 1: Genotyping 2](#_Toc159237734)

[Supplement 2: Descriptives 3](#_Toc159237735)

[Supplement 3: Supplemental Figure 4](#_Toc159237736)

**Supplement 1: Genotyping**Data were genotyped on the Affymetrix 6.0, Axiom and Illumina GSA panels following manufacturers protocols (REF). For each individual platform, DNA samples were checked for gender mismatches, heterozygosity with Plink F value being in between -0.10 to 0.10 and Plink estimated Identity By Descent (IBD) mismatches in comparison to the known family structure. For call rate in the samples, each sample needed to have at least 90% genotyped and furthermore, at least 80% of the genotypes needed to be present on each separate chromosome 1-22 plus X for each person. All problematic samples were removed given all above criteria.
 The SNP quality control was based on the following filters applied in each platform: call rate should be over 95%, Hardy Weinberg p-value should be over 0.0001, Minor allele frequency should be over 0.01 and Mendelian error rate should be less than 1%. Based on several plate control samples in Affymetrix 6 (n=4 typed 38-84 times) and Axiom (n=2 typed 33-37 times) and the at least 2 times typed samples on the GSA platform sets, SNPs were removed if the genotypes differed more than 1% between multiple measurements.
 SNP alignment was done to the positive strand of build 37 (HG19) and only overlapping SNPs with an allele frequency being less than 0.20 apart from the reference (1KG only EUR) panels were selected. Palindromic SNPs with an allele frequency of 0.40-0.60 were removed. After this alignment of the data, the platforms are also inherently aligned with each other. Therefore, at this point the data of the 3 platforms were merged into a single dataset and based on the overlapping SNPs in platforms, IBD was re-checked against the known family structure (now across platforms). Mismatching IBD samples as compared to known family structure were removed. A couple of samples which had discordant DNA across platforms were removed in this step. Samples were selected for each participating individual in the following order: Axiom, Affymetrix 6.0 and then Illumina GSA. The array with the smallest sample size was selected first, to keep this set the largest for imputation. Data were converted to VCF format with Plink. 
 The NTR data were imputed with Beagle 5.4 against the 1000 genomes Phase3 V5A (1KG) panel. For the 1KG panel the VCF data were straight converted to Bref3 format. After imputation, the resulting VCF data of the 3 platforms were merged into single chromosome sets 1-22 plus X using BCFtools. With QCtool version 2.20 these data were then also converted to BGEN format, and buest-guess genotypes using Plink 1.9. 
 Twenty 1000 genomes projected principal components for the genotype data were calculated from the 1KG imputed SNPs, which were also present on at least one platform using the EIGENSTRAT smartpca tool. For this analysis the mixed genotyped and imputed SNPs were filtered based on MAF>0.05, HWE p > 0.001, call rate > 0.98, Mendelian error rate < 1% and imputation info>=90%. These SNPs were subsequently pruned with Plink (option --indep 50 5 2) and SNPS in long range LD blocks were removed as described in (PMID: 23531865). This left 110558 SNPs for PC analysis. From the 1000 genomes reference panel all samples with the same SNPs were selected and then merged with the NTR data. Subsequently PCs were calculated in the 1000 genomes set, and then projected upon the NTR data with the smartpca software.

**Supplement 2: Descriptives**

| **Supplemental Table 1: Descriptives** | | | |
| --- | --- | --- | --- |
| **Variable** | **N** | **Mean** | **SD** |
| Self-control | 7090 | 2.47 | 2.21 |
| SLE previous year | 7090 | 0.42 | 0.72 |
| SLE lifetime | 7090 | 2.45 | 1.86 |
| PGI ADHD | 7090 | .00 | .00 |
| PGI Aggression | 7090 | .00 | .00 |
| Age | 7090 | 41.20 | 15.40 |
| Sex | 7090 | 66% female |  |
| *Note:* There were no significant mean level differences across women and men in self-control (t(4744)=-.03, *p*=.80). There was a significant association between self-control and age, with older participants showing higher average self-control levels (*r*=-.14, *p*<.001). There were no significant sex differences in the experience of life stressors last year (t(4943)=-2, *p*=.05) or across the lifetime (t(4871)=0.3, *p*=.80). There was a significant association between age and stressful life events: older participants were more likely to have experienced stressful life events across their life (*r*=.31, *p*<.001) but less likely to have experienced it in the last year (*r*=-.09, *p*<.001). | | | |

**Supplement 3: Supplemental Figure**

| 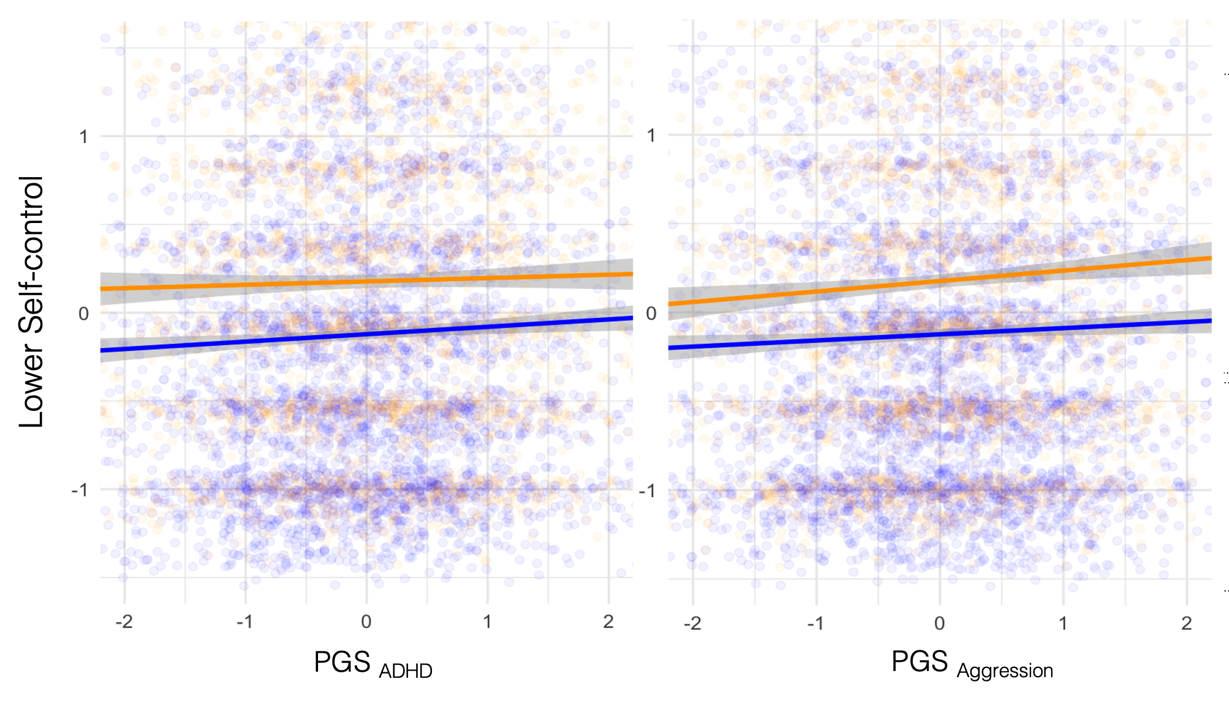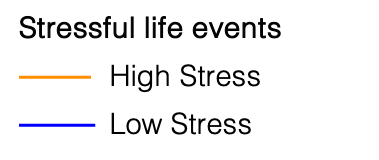 |
| --- |
| **Supplemental Figure 1.** Association between PGS for self-control problems and self-control  *Note:* For illustration purposes, participants were stratified into a “high stress” and “low stress” group bymean split of stressful life events experienced last year (M_stressfull life events_= 0.40). The lines for the two groups are approximately parallel and resemble those in Panel C in **Figure 1**, indicating there is a main effect of stressful life events and genetic propensity on self-control problems, respectively, but no significant interaction effect between the two. See **Figure 2** in the main manuscript for plots for life events experienced across the lifetime. |
